# Genetic and immunologic characteristics of colorectal cancer patients with KRAS mutations and predictive significance of tumor immune microenvironment in adjuvant chemotherapy

**DOI:** 10.1016/j.gendis.2023.05.002

**Published:** 2023-06-19

**Authors:** Zhengqi Wen, Wenliang Li, Chengmin Shi, Junrui Ma, Sihui Zhao, Ruize Zhou, Xihong Liu, Rong Yang, Zhiping Zhang, Hushan Zhang, Bo Li

**Affiliations:** a Department of Oncology, The First Affiliated Hospital of Kunming Medical University, Kunming, Yunnan 650032, China; b The Third Affiliated Hospital of Kunming Medical University (Yunnan Cancer Center), Kunming, Yunnan 650021, China; c Gastrointestinal and Hernia Surgery, The First Affiliated Hospital of Kunming Medical University, Kunming, Yunnan 650032, China; d School of Nursing, Yunnan University of Chinese Medicine, Kunming, Yunnan 650022, China; e Department of General Surgery, The First People's Hospital of Yunnan Province, Kunming, Yunnan 650000, China; f Department of General Surgery, The Affiliated Hospital of Yunnan University, Yunnan 650011, China; g Medical Department, 3D Medicines Inc., Shanghai 201114, China; h Zhaotong Health Vocational College, Zhaotong, Yunnan 657000, China

Colorectal cancer (CRC) is one of the most common cancers around the world, and it is one of the leading causes of cancer-related death.^1^ Although anti-EGFR therapy and immune checkpoint inhibitor (ICI) therapy are becoming more and more important for colorectal therapy, however, clinical outcomes of current treatments for metastatic CRC, especially with ICIs, have been shown to be affected by the status of KRAS. Therefore, KRAS mutation status detection has become a very important diagnostic factor for managing metastatic CRC patients. There have also been a lot of immunotherapy explorations in CRC, such as clinical trials of pembrolizumab and nivolumab. The clinical trial Keynote 177 is a groundbreaking research in CRC, which evaluated the efficacy of programmed death 1 blockade as first-line therapy for MSI-H-dMMR advanced or metastatic CRC. However, the results of subgroups revealed that immunotherapy did not benefit more than chemotherapy in patients with KRAS mutation.^2^ While the mechanism behind these clinical results keeps unclear, herein, we comprehensively analyzed the genetic and immunologic characteristics of CRC with KRAS mutation, and try to explore the mechanisms and potential predictive biomarkers, especially from the prospect of tumor immune microenvironment (TIME).

KRAS is one of the most famous oncogenes.^3^ As all known, the status of KRAS gene is closely related to the prognosis of CRC patients and their response to treatments including anti-EGFR treatment, ICI therapy, and even chemotherapy; therefore, more about KRAS mutation should be put into account for therapy choice and management of CRC patients. Based on data from 5260 primary CRC (Fig. 1A), the genetic and immunologic landscape of Chinese CRC patients with KRAS mutation was evaluated, and the clinical significance of TIME was explored and excavated. Firstly, the proportion of Chinese CRC patients with KRAS mutation was calculated. As the pie chart shows, KRAS mutation account for 47.11% of Chinese CRC patients (Fig. 1B). In addition to dMMR/MSI-H, tumor mutational burden and programmed death ligand 1 have also been demonstrated to have the potential to screen patients who can benefit more from ICIs.^4^ Therefore, in this research, the proportion of MSI-H, the level of tumor mutational burden, and the expression of programmed death ligand 1 in CRC patients with and without KRAS mutation were analyzed first. As results showed, no significant differences among these biomarkers were found between CRC patients with and without KRAS mutation (Fig. 1C–E; Fig. S1). It may infer that other prospects should be put into consideration to better know the potential mechanism of different responses to treatments and different clinical outcomes shown in CRC patients with and without KRAS mutation.

**Figure 1:**
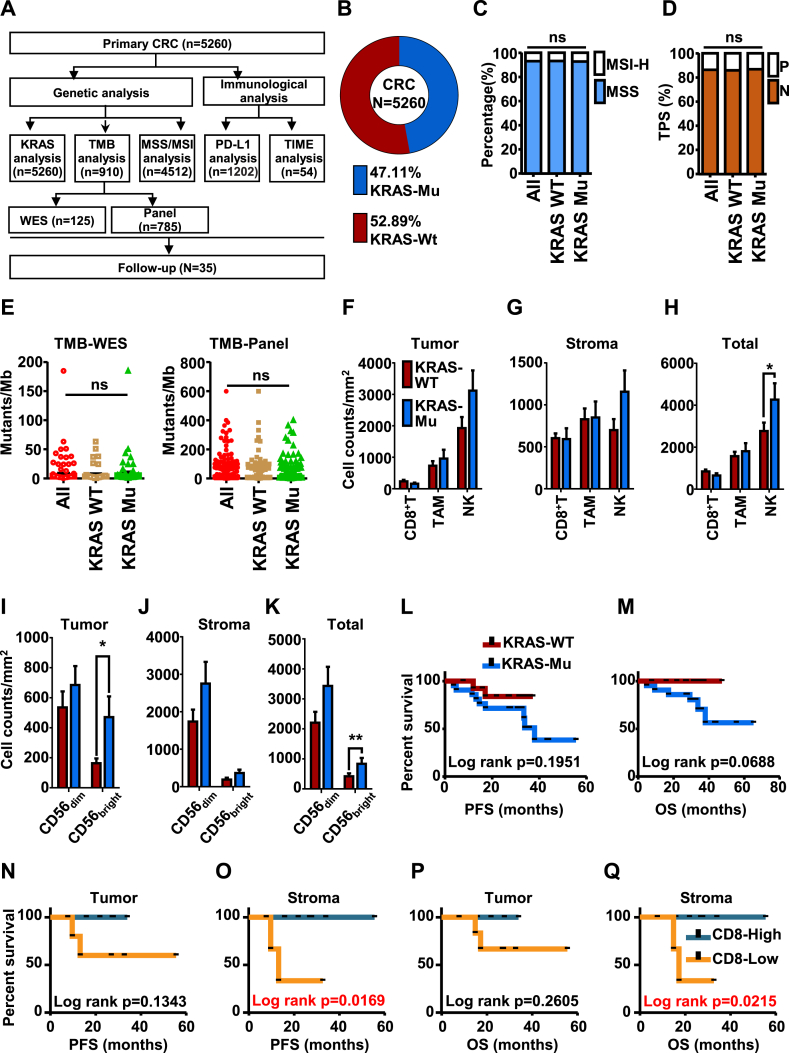
Genetic and immunologic characteristics of CRC patients with and without KRAS mutation and the predictive significance of TIME in CRC patients. **(A)** Flow charts for data analysis in this research. **(B)** The frequencies of KRAS mutations in Chinese CRC patients. **(C)** Comparison of MSI-H between CRC patients with and without KRAS mutation. **(D)** Comparison of tumor programmed death ligand 1 (PD-L1) expression between CRC patients with and without KRAS mutation in different exons. PD-L1 expression was quantified as negative (TPS < 1%), intermediate positive (1% ≤ TPS < 49%), and strong positive (TPS ≥ 50%) for available cases and tabulated across different KRAS mutation subtypes. **(E)** Whole exome sequencing- and panel-calculated tumor mutational burden (TMB) levels in CRC patients with and without KRAS mutation. **(F–K)** Analysis of immune cell infiltration in tumor (intra-tumoral region) and stroma of 54 CRC patients. Immune cell counts both in tumor (intra-tumoral region) and stroma of 54 CRC patients with (Mu) and without (wild type, WT) KRAS mutation. The counts of CD8^+^ T cells, NK cells, and tumor-associated macrophages (TAM) in tumor **(F)**, stroma **(G)**, and tumor plus stroma **(H)** were compared between the patients with and without KRAS mutation. Percentages of CD56_bright_ and CD56_dim_ NK cells in tumor **(I)**, stroma **(J)**, and tumor plus stroma **(K)** were compared between the patients with and without KRAS mutation. **(L**–**Q)** Analysis of predictors of adjuvant chemotherapy for CRC patients. Kaplan–Meier estimates for progressive free survival (PFS) (L, N, O) and overall survival (OS) (M, P, Q). Patients were stratified according to KRAS status (L, M), infiltration of CD8^+^ T cells in the tumor (N, P) and stroma (O, Q). ^∗^*P* < 0.05, ^∗∗^*P* < 0.01, ^∗∗∗^*P* < 0.001, ^∗∗∗∗^*P* < 0.0001.

Here we assessed characteristics of TIME in patients with or without KRAS mutation, to find out the underlying mechanisms especially that prevent KRAS-mutated CRC patients from benefiting from immunotherapy, and try to find the potential direction to transform these patients to be immunotherapy responders.

In total, 54 CRC patients whose tumor tissues were available for TIME analysis by the technology of multiplex immunofluorescence (Fig. S2A). First, we evaluated differences in immune cells infiltration, including CD8^+^ T cells, tumor-associated macrophages (TAM), and natural killer (NK) cells, both in tumor region and stroma of the tumor tissue samples (Fig. 1F–K; Fig. S2, 3). The absolute number of these immune cells infiltrated in TIME was analyzed, and results found that more NK cells in the stroma plus tumor region in the group of KRAS mutation than the wild type (WT) (Fig. 1H). The percentage of NK cells in the KRAS mutant group was higher than in the wild type (WT) regardless of in stroma, tumor, or stroma plus tumor (Fig. S2B–D). Then, we further analyzed the sub-population cells of TAM and NK cells, which were M1 (marked as CD68^+^ HLA-DR^+^), M2 TAM (marked as CD68^+^ HLA-DR^−^) (Fig. S3), CD56_bright_ NK cells, and CD56_dim_ NK cells. Analysis of the percentage and the absolute number of these cells revealed that only CD56_bright_ NK cells were higher in both tumor and tumor plus stroma in the group of KRAS mutation than in the WT (Fig. 1I, K; Fig. S2E–G). For M1 and M2 TAM, no significant differences were found both in percentage and absolute cell number counts (Fig. S3).

Our results showed more CD56_bright_ NK cells were found in KRAS mutated patients (Fig. 1F–K; Fig. S2). This may suggest that too much CD56_bright_ NK cell infiltration was related to the poor response to ICIs. It was demonstrated that CD56_bright_ cells showed lower cytotoxic activity than CD56_dim_ NK cells; this kind of CD56_bright_ cells contain fewer granzymes, perforin, and cytolytic granules. Besides, in this research, we analyzed the TIME profile of a KRAS-mutant patient treated with an ICI. Although higher CD8^+^ T cells infiltrated in this TIME of this patient, this patient showed poor response to sintilimab, which may be related to the higher infiltration of CD56_bright_ NK cells in the TIME in this patient (Fig. S4).

Besides, the prognostic and predictive efficacy of TIME has been widely demonstrated in several solid tumors.^5^ We retrospectively collected and analyzed the follow-up data of 35 CRC patients who received adjuvant chemotherapy after surgery, the baseline characteristics of these patients were displayed in Table S1. Both progressive free survival and overall survival were analyzed among these patients, and results showed that no significant differences were found between patients with and without KRAS mutation (Fig. 1L–Q). The difference in prognosis between CRC patients with and without KRAS mutation was compared first, while the results showed no significant difference (Fig. 1L, M). The predictive efficacy of TIME for adjuvant chemotherapy was further analyzed. Log-rank progressive free survival and overall survival analysis were performed by using cutoff values of the median density of tumor-infiltrating immune cells, such as CD8^+^ T cells (CD8^+^ T-high patients were defined as patients with CD8^+^T cell count above the median of CD8^+^ T cell density in all these samples that available for TIME analysis, others defined as CD8^+^ T-low). As results showed, better progressive free survival and overall survival were found in patients with CD8^+^ T-High in stroma than patients with CD8^+^ T-low (Fig. 1O, Q). However, other members in the TIME showed no predictive significance for adjuvant chemotherapy in CRC patients (Fig. S5, 6).

In this study, we analyzed the difference in TIME in CRC patients with and without KRAS mutations, to explore the potential role of TIME in KRAS-mutated CRC patients, therefore, it may help to improve the clinical benefit of ICIs in KRAS-mutated CRC by regulating and optimizing TIME in the future clinical practice. In another hand, we explored the role of TIME in predicting CRC patients' survival after adjuvant chemotherapy. Our results displayed CD8^+^ T cells may predict better benefits from adjuvant chemotherapy.

In conclusion, more details and characteristics of KRAS mutation should be put into consideration for treatment strategy choice, including mutation in different exons and immune cell infiltration. The different responses to ICI therapy for CRC patients with or without KRAS mutation are mainly associated with factors of TIME, especially CD56_bright_ NK cells, which suggested that the clinical benefit of ICIs for CRC patients with KRAS could be well improved through the regulation of NK cells, especially, CD56_bright_ NK cells. In addition, for postoperative patients, especially those requiring adjuvant therapy after surgery, TIME could be used to predict the benefit of adjuvant chemotherapy, and based on the detection of TIME, such CD8^+^ T cells infiltrated in the tumor could screen patients more suitable for adjuvant chemotherapy.

## Ethics declaration

The study was reviewed and approved by the Ethics Committee of The Affiliated Hospital of Yunnan University, and the Ethics Committee of The First Affiliated Hospital of Kunming Medical University, Yunnan Province, China. Written informed consent was obtained from all the patients whose tumor tissues were subjected to next-generation sequencing detection and tumor immune microenvironment detection.

## Author contributions

Zhengqi Wen, Wenliang Li, and Hushan Zhang put forward the content of the paper. Hushan Zhang wrote the manuscript. Hushan Zhang, Zhengqi Wen, Wenliang Li, and Chengmin Shi prepared all figures. Hushan Zhang and Bo Li analyzed all data. Junrui Ma, Sihui Zhao, Ruize Zhou, Xihong Liu, Rong Yang, and Zhiping Zhang reviewed the literature and collected the clinical data. All authors read and approved the final manuscript.

## Conflict of interests

The authors declare no potential conflict of interests.

## Funding

This research was supported by the 10.13039/501100001809National Natural Science Foundation of China (No. 81960100, 81760511).

## Figures and Tables

**Fig. S1:**
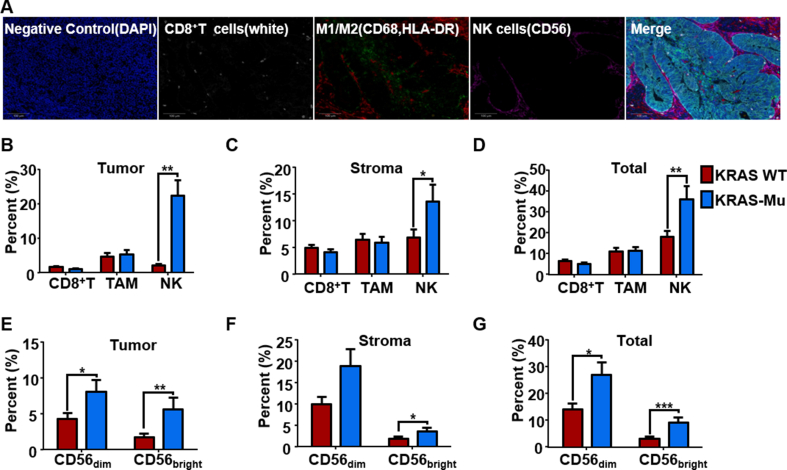


**Fig. S2:**
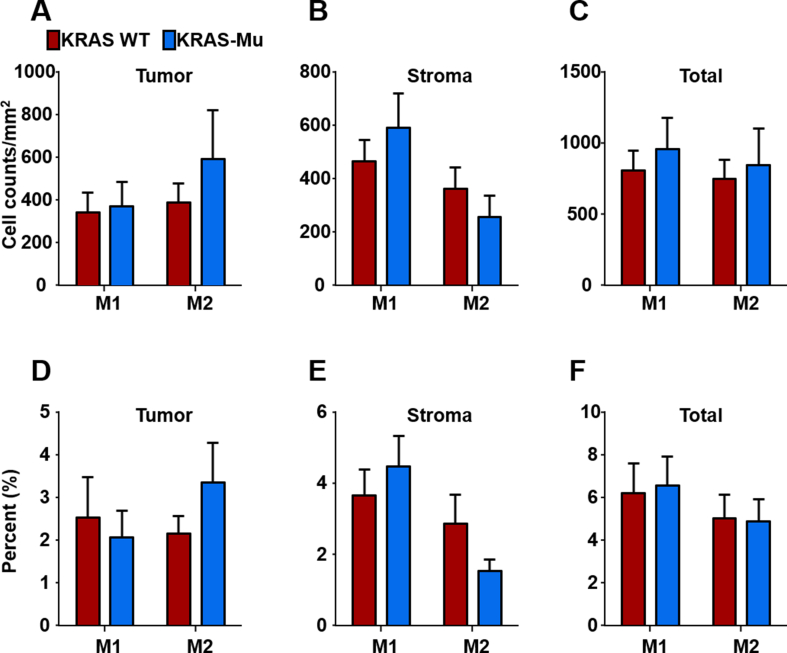


**Fig. S3:**
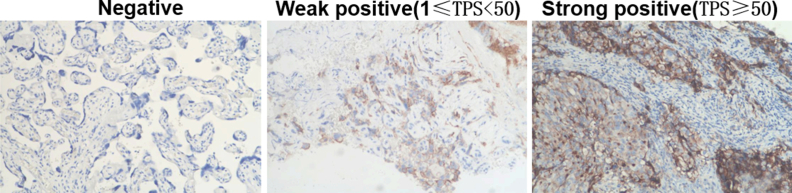


**Fig. S4:**
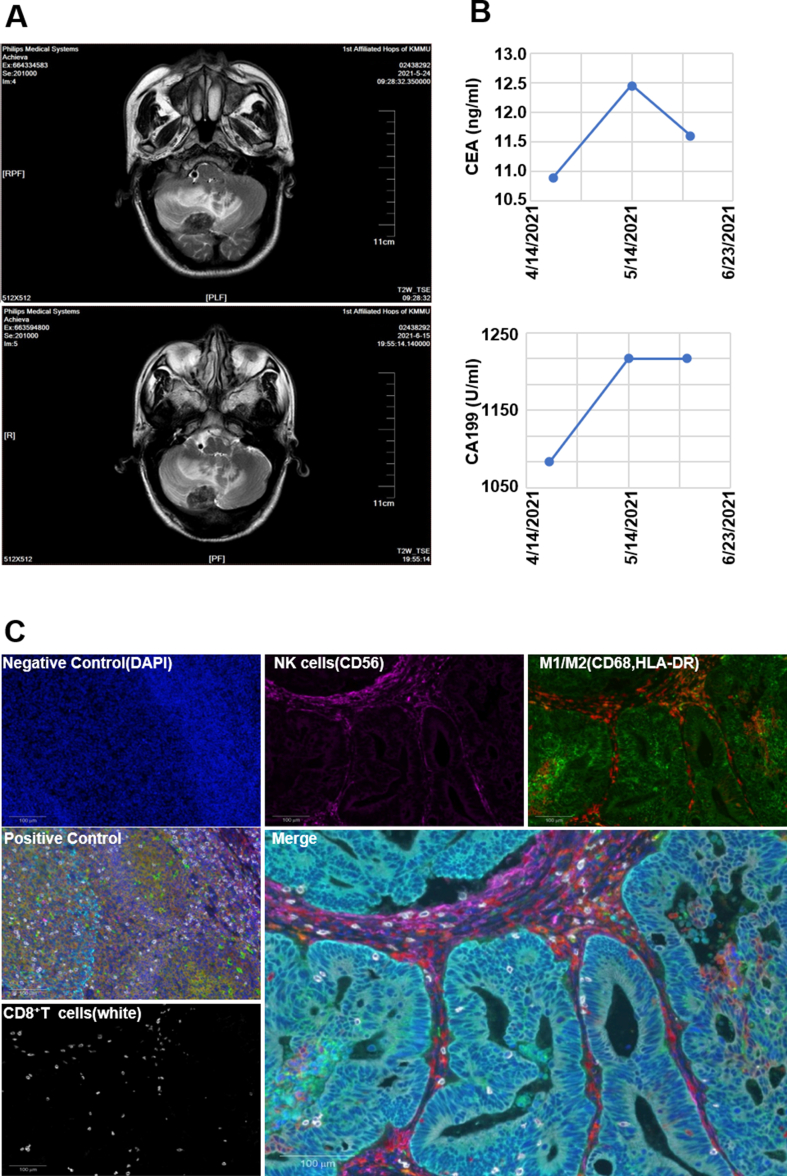


**Fig. S5:**
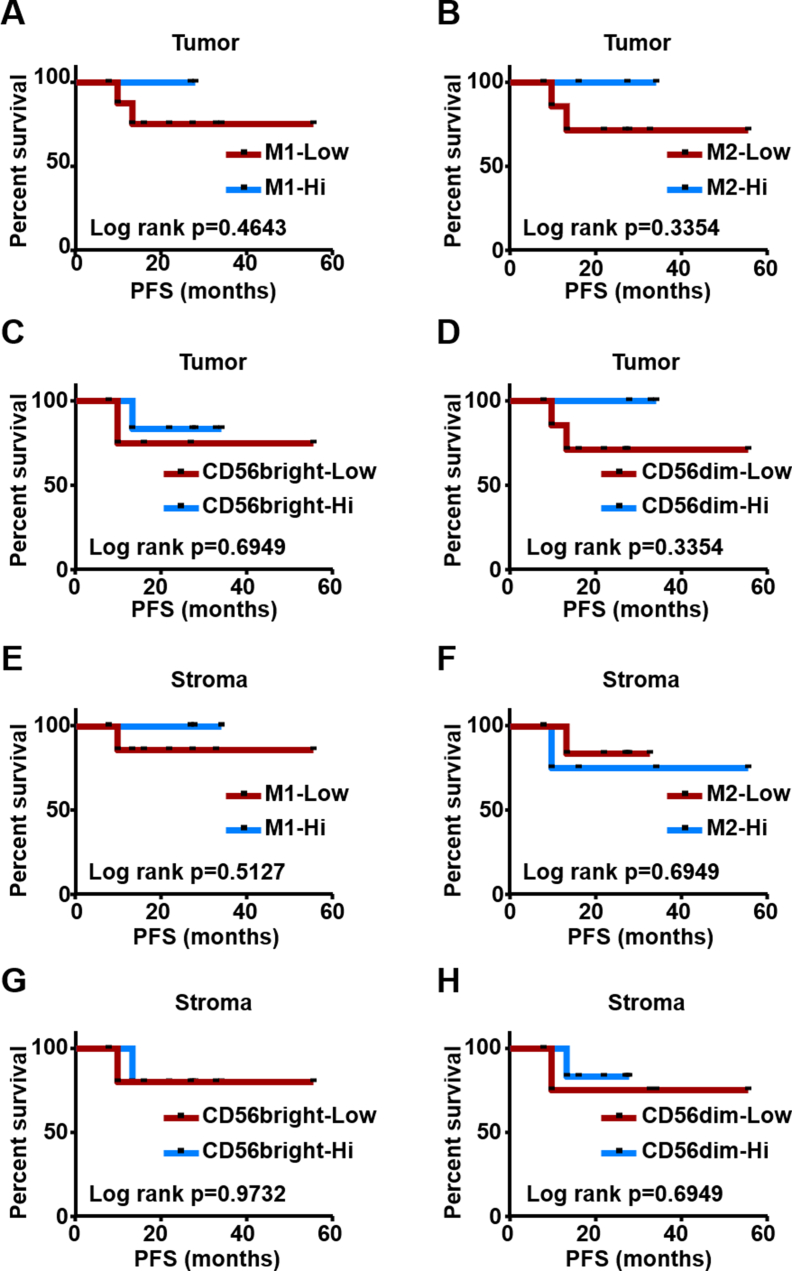


**Fig. S6:**
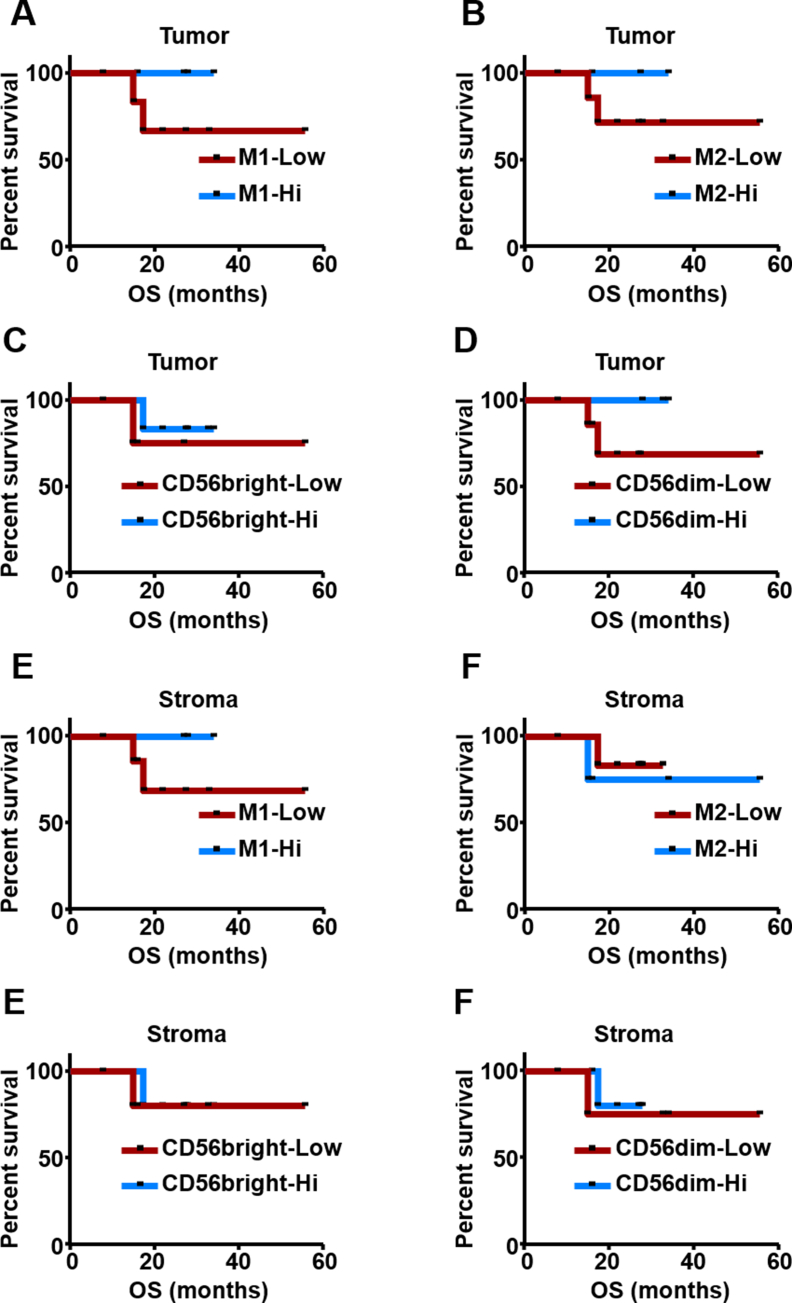


## References

[1] Roncucci L., Mariani F. (2015). Prevention of colorectal cancer: how many tools do we have in our basket?. Eur J Intern Med.

[2] André T., Shiu K.K., Kim T.W. (2020). Pembrolizumab in microsatellite-instability–high advanced colorectal cancer. N Engl J Med.

[3] Liu P., Wang Y., Li X. (2019). Targeting the untargetable KRAS in cancer therapy. Acta Pharm Sin B.

[4] Yi M., Jiao D., Xu H. (2018). Biomarkers for predicting efficacy of PD-1/PD-L1 inhibitors. Mol Cancer.

[5] Zhou Y., Tian Q., Wang B.Y., Yang J., Zhao S.D., Yang J. (2021). The prognostic significance of TILs as a biomarker in triple-negative breast cancer: what is the role of TILs in TME of TNBC?. Eur Rev Med Pharmacol Sci.

