# Supplementary material for: Genetic and immunologic characteristics of colorectal cancer patients with KRAS mutations and predictive significance of tumor immune microenvironment in adjuvant chemotherapy
Source: Genes Dis. 2023 Jun 19;11(3):100983. doi: 10.1016/j.gendis.2023.05.002 (PMC10825235; doi:10.1016/j.gendis.2023.05.002)
Supplement: Multimedia component 1 [file mmc1.docx]

**Supplementary Materials**

Page Number

Supplementary Figure 1 2

Supplementary Figure 2 2

Supplementary Figure 3 3

Supplementary Figure 4 4

Supplementary Figure 5 6

Supplementary Figure 6 7

Supplementary Table 1 8

Supplementary Table 2 9

Supplementary Materials and Methods 11-12

**Figure S1**

**
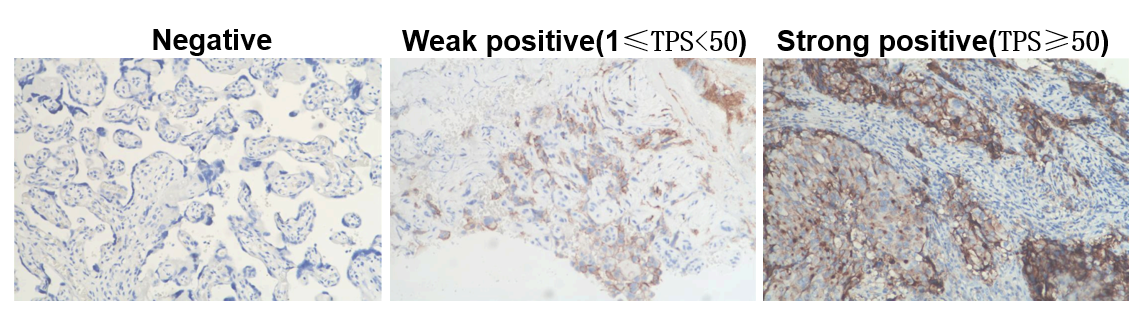
**

**Figure S1**. PD-L1 expression level was evaluated by IHC. PD-L1 expression was quantified as negative (TPS<1%), intermediate positive (1%≤TPS<49%), and strong positive (TPS≥50%) for available cases and tabulated across different KRAS mutation subtypes. Representative staining of PD-L1 expression through IHC (200X).

**Figure S2**

**
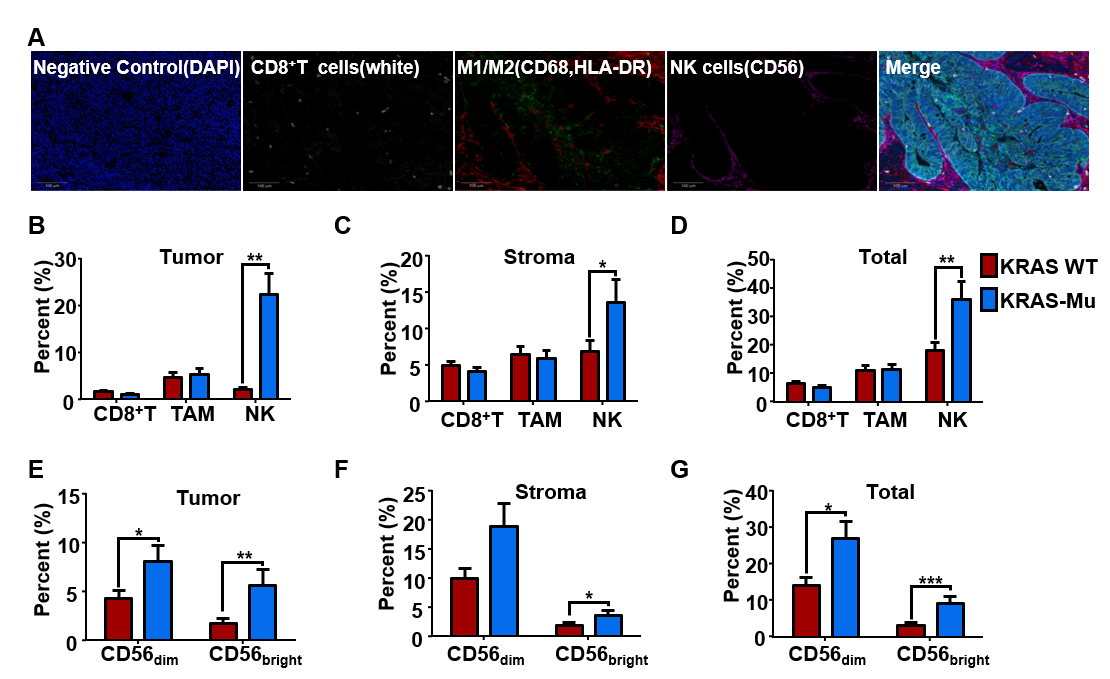
**

**Figure S2**. Immune cell counts both in tumor (intra-tumoral region) and stroma of 54 CRC patients with and without KRAS mutation. **(A),** Representative staining for CD8+T cells, CD56bright and CD56dim NK cells, CD68+HLA DR+ M1, CD68+HLA-DR- M2 TAM. CD8 (white), CD56 (purple), CD68 (green), HLA-DR (red), panCK/S100(cyan), DAPI (blue). Numbers of CD8+T, NK cells, tumor associated macrophages (TAM) in tumor **(B)**, stroma **(C)**, in total of tumor plus stroma **(D)** were compared between with and without KRAS mutation groups. Numbers of CD56bright, CD56dim NK cells in tumor **(E)**, stroma **(F)**, in total of tumor plus stroma **(G)** were compared between with and without KRAS mutation groups (KRAS-Mu and KRAS-WT). * means p<0.05,** means p<0.01,*** means p<0.001,**** means p<0.0001.

**Figure S3**

**
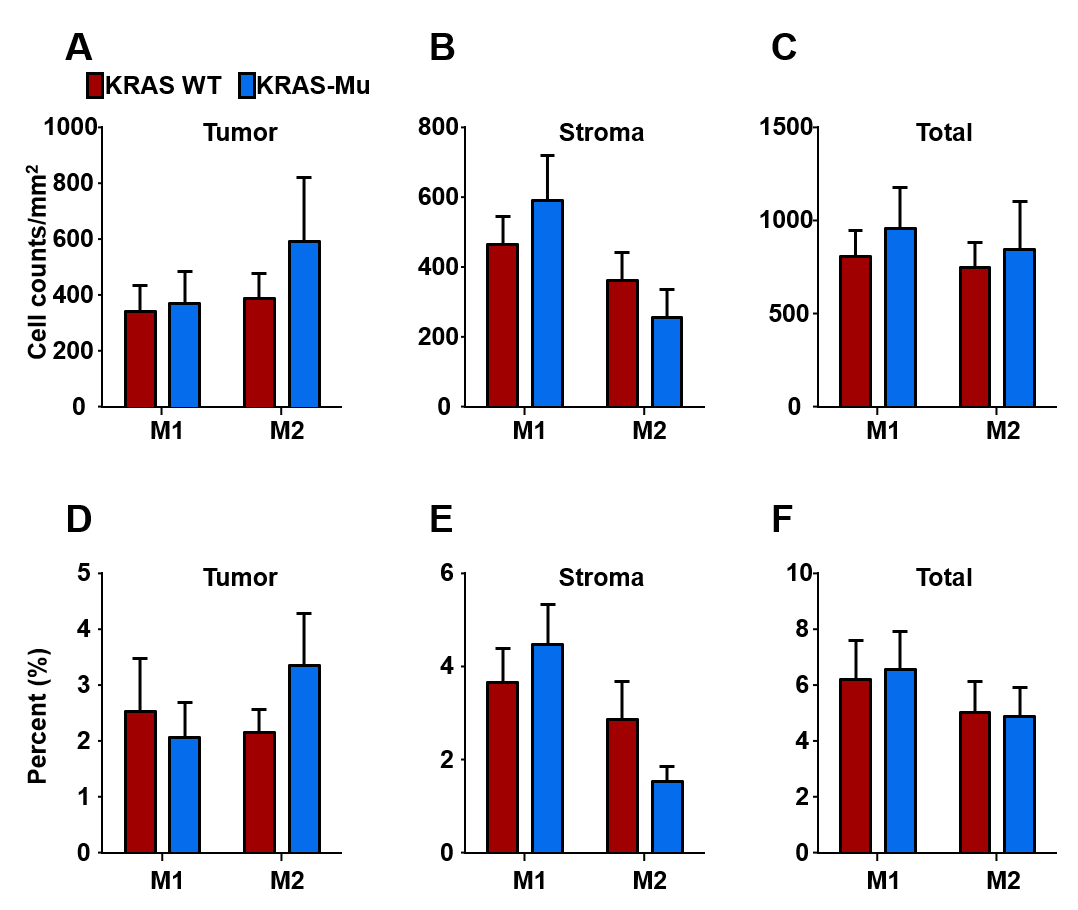
**

**Figure S2**. Cell counts and percentage of TMA in tumor immune microenvironment (TIME) in CRC patients with and without KRAS mutations.

Analysis of cell counts **(A-C)** and percentage **(D-F)** of TAM subsets cells (M1 and M2) in tumor (intra-tumoral region) **(A, C)**, stroma **(B, E)** and in total of tumor plus stroma of CRC patients **(C, F)**.

**Figure S4**

**
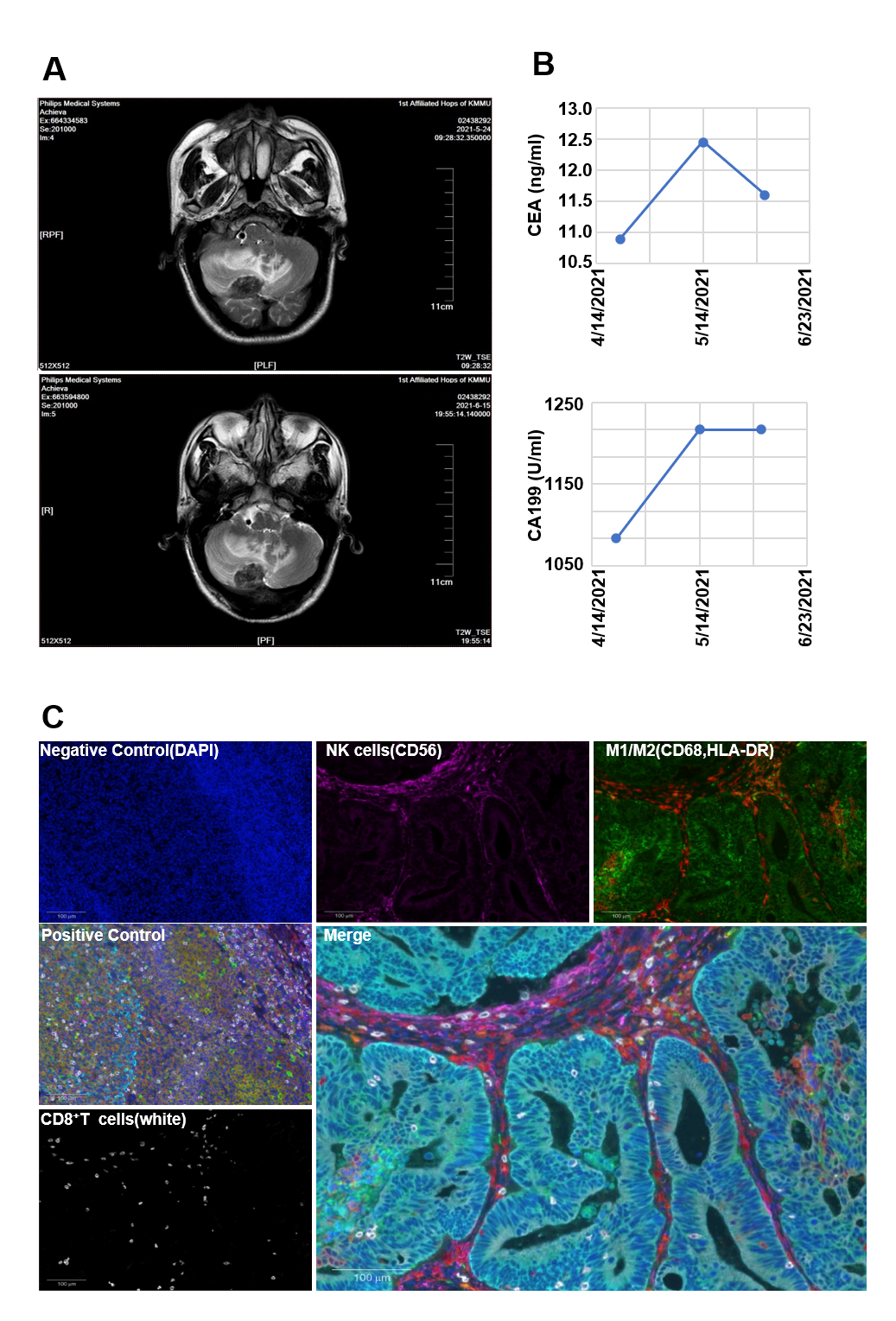
**

**Figure S4**. Representative case presentation. A 68-year-old man with rectal cancer who recurred after one year of surgical treatment, received therapy of immune checkpoint inhibitor after progression on standard therapy, that was sintilimab, (200 mg sintilimab was intravenously administered on D1 once every 3 weeks), a monoclonal antibody against PD-1, as well as supportive care such as fluid rehydration and nutrition. After two months of treatment, the disease progressed and brain metastases appeared **(A-B)**. Genetic testing revealed that the patient had a KRAS mutation (KRAS G12A). The results of the immune microenvironment detected by mIF showed that the patient had higher CD8+ T cell infiltration (higher than the median value of CD8+ T cell infiltration in CRC patients in this research), and high CD56_bright_ NK cell infiltration (higher than the median value of CD56_bright_ NK cells infiltration in CRC patients in this research) **(C)**.

**(A)**, computed tomography of brain since initial of sintilimab treatment, during the treatment and after disease progression. (**B)**, level of CEA and CA199 of the patients c since initial of sintilimab treatment, during the treatment and after disease progression. **(C)**, TIME of the patients.

**Figure S5**

**
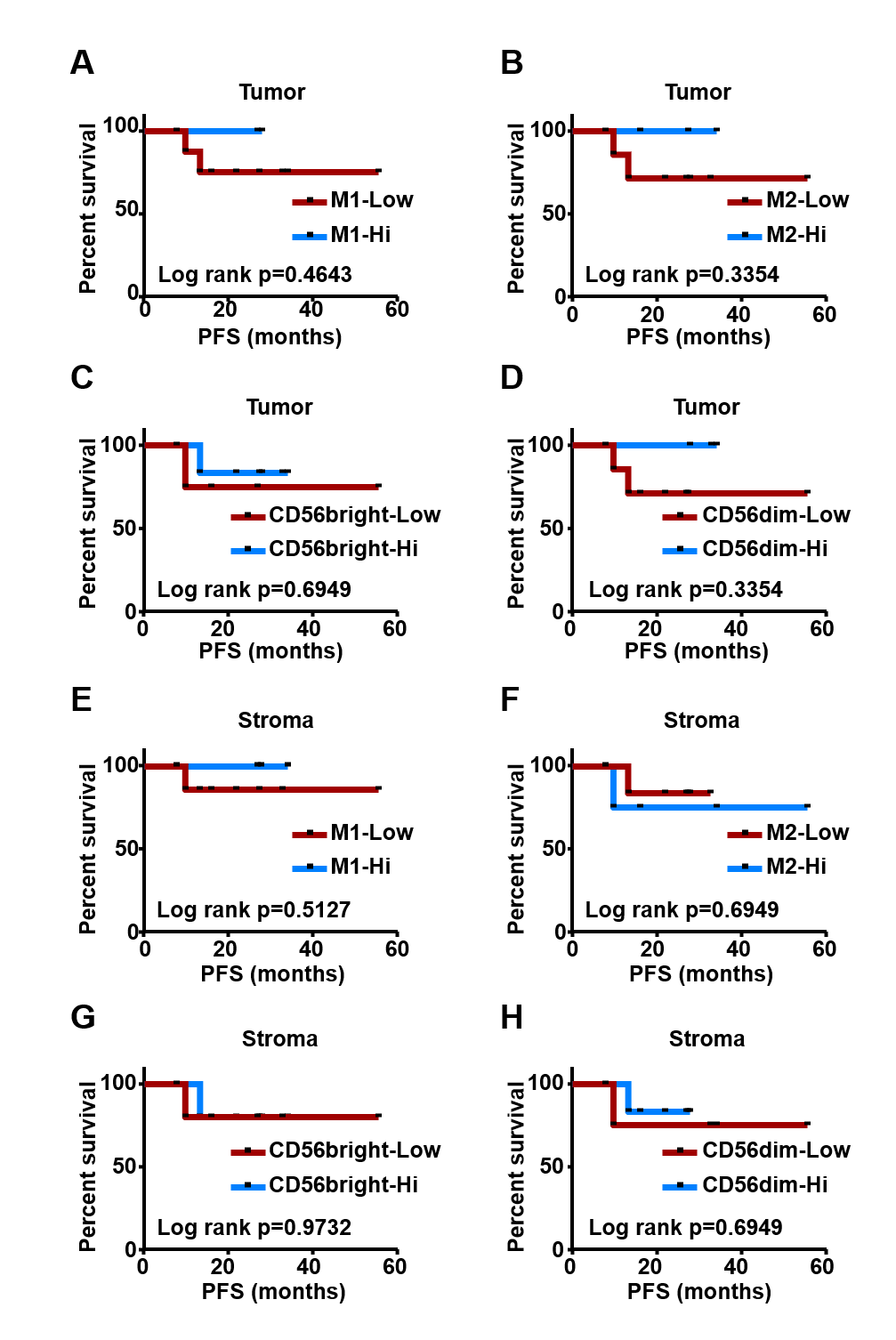
**

**Figure S5**. Analysis of TIME as predictors of PFS for adjuvant chemotherapy in CRC patients.

Kaplan Meier estimates for PFS; patients were stratified according to infiltration of M1, M2, CD56bright NK, CD56dim NK cells in tumor **(A- D)** and stroma **(E-H)**.

**Figure S6**

**
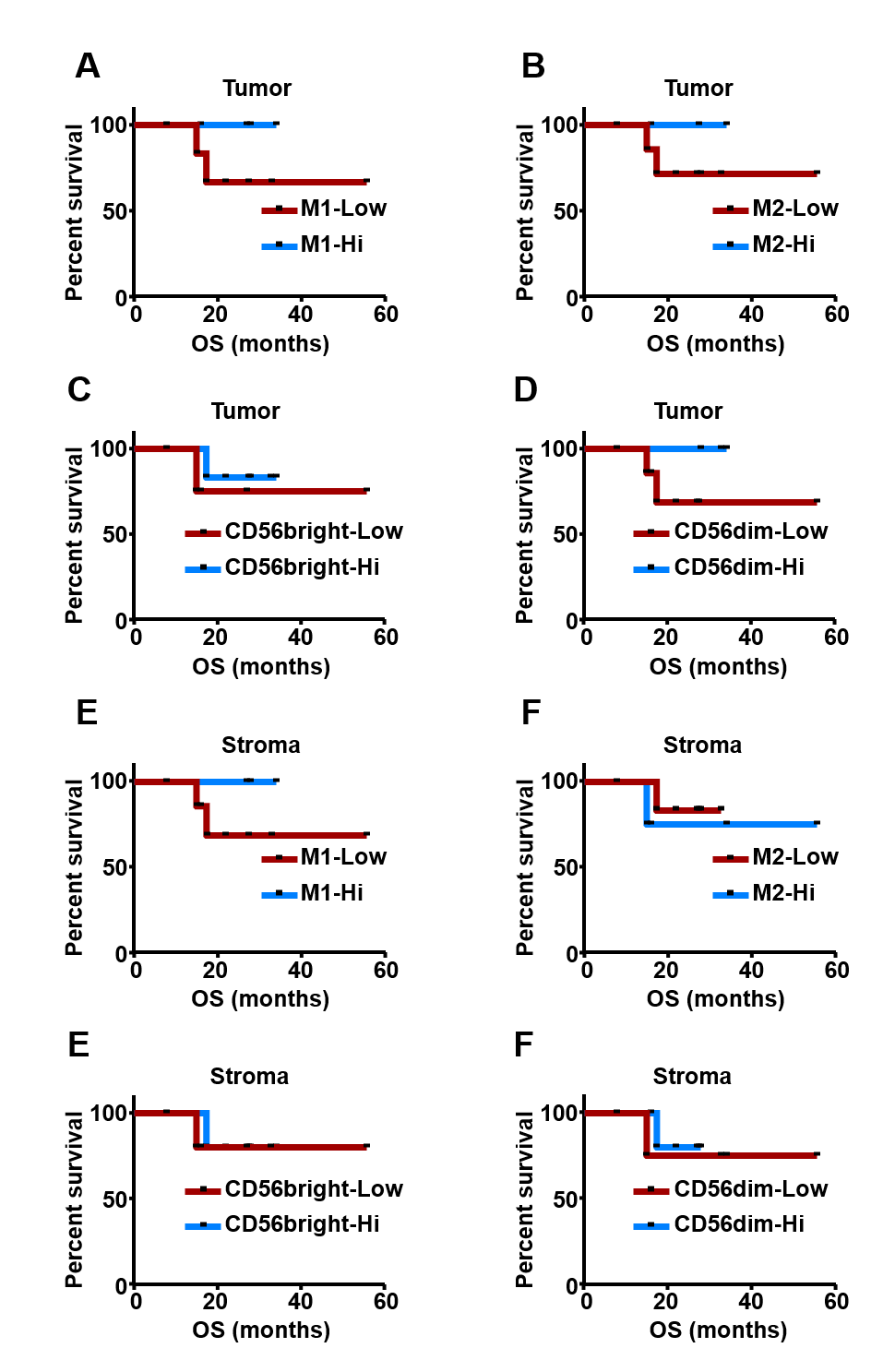
**

**Figure S6**. Analysis of TIME as predictors of OS for adjuvant chemotherapy in CRC patients.

Kaplan Meier estimates for OS; patients were stratified according to infiltration of M1, M2, CD56bright NK, CD56dim NK cells in tumor **(A-D)** and stroma **(E-H)**.

**Table S1. Patient characteristics in the follow-up cohort.**

| **Characteristics** | **N** | **%** |
| --- | --- | --- |
| **Total** | 35 |  |
| **Gender** |  |  |
| Male | 22 | 62.86 |
| Female | 13 | 37.14 |
| **Age** |  |  |
| ≥60 | 23 | 65.71 |
| <60 | 12 | 34.29 |
| **Location** |  |  |
| Left colon cancer | 13 | 37.14 |
| Right colon cancer | 20 | 57.14 |
| Rectal cancer | 12 | 34.29 |
| **Stage** |  |  |
| II | 4 | 11.43 |
| III | 31 | 88.57 |
| **KRAS mutation** |  |  |
| Without (KRAS WT) | 14 | 40.00 |
| With (KRAS Mu) | 21 | 60.00 |
| **Available TIME analysis** | 11 | 31.43 |
| **Adjuvant therapy** |  |  |
| FOLFOX (6 month) | 20 | 57.14 |
| Capecitabine | 4 | 11.43 |
| CAPEOX (3 month) | 11 | 31.43 |

**Table S2. List of genes of the 733-gene panel**

| ABL1 | CDX2 | FGFR4 | MLH1 | PTEN | VEGFA | JMJD1C | TRIM37 | BCL11A | EZR | TBL1XR1 | PLXNB1 | LIG1 | RNF168 | POLD3 |
| --- | --- | --- | --- | --- | --- | --- | --- | --- | --- | --- | --- | --- | --- | --- |
| ACVR2A | CHD2 | FH | MLLT3 | PTK6 | VHL | LMO1 | TSHR | BCL11B | FAT4 | TCF7L2 | SPRED1 | LIG3 | RNF4 | POLD4 |
| AFF3 | CHEK1 | FHIT | MPL | PTPRD | NSD3 | LZTR1 | UROD | BCORL1 | FUBP1 | TCL1A | ERF | LIG4 | RNF8 | POLE2 |
| AKT1 | CHEK2 | FLCN | MRE11A | RAC1 | ZNF479 | MAX | WAS | BIRC3 | FUS | TET1 | RPS6KA3 | MAD2L2 | RPA1 | POLE4 |
| AKT2 | CHIC2 | FLT1 | MSH2 | RAD50 | ZNRF3 | MEN1 | WRN | BRD4 | GAS7 | TFE3 | GSK3B | MBD4 | RPA2 | PPP4R1 |
| AKT3 | CIC | FLT3 | MSH3 | RAD51 | ABCB11 | MTAP | WT1 | CACNA1D | H3F3A | TNFAIP3 | NOTCH3 | MDC1 | RPA3 | PPP4R3A |
| ALK | CIITA | FLT4 | MSH6 | RAD51C | APOBEC3B | MUTYH | XPA | CALR | HIF1A | USP8 | NOTCH4 | MGMT | RPA4 | PPP4R3B |
| ANK1 | CRBN | FOXA1 | MTOR | RAF1 | AXIN2 | NBN | XPC | CAMTA1 | HIP1 | WIF1 | ALKBH2 | MLH3 | RRM2B | PPP4R4 |
| APC | CRLF2 | FRS2 | MYC | RARA | BARD1 | NHP2 | XRCC2 | CANT1 | HNRNPA2B1 | XPO1 | ALKBH3 | MMS19 | SETMAR | RAD9B |
| AR | CRNKL1 | G6PD | MYCN | RB1 | BMPR1A | NME1 | HOXB13 | CARD11 | HOXA11 | ZFHX3 | APEX1 | MNAT1 | SEM1 | RBX1 |
| ARAF | CRTC3 | GATA3 | MYD88 | RET | BUB1B | NOP10 | BCL2L1 | KNL1 | IL6ST | ACVR1B | APEX2 | MPG | SHPRH | RFC1 |
| AREG | CSF1R | GLI2 | NF1 | RGS7 | CDC73 | NTHL1 | BCL6 | CASP8 | KDM6A | ARID1B | CENPS | MSH4 | SMUG1 | RFC2 |
| ARHGAP5 | CSF3R | GNA11 | NF2 | RICTOR | CDKN1C | PHOX2B | CDK8 | CBFA2T3 | KEAP1 | DNMT1 | APLF | MUS81 | SPO11 | RFC3 |
| ARID1A | CTNNB1 | GNAQ | NFE2L2 | RNF43 | CEBPA | PMS1 | FOXP1 | CBFB | KLF4 | FOXL2 | APTX | NEIL1 | TDG | RFC4 |
| ARNT | CTNND2 | GNAS | NFIB | ROS1 | COL7A1 | POLH | GRIN2A | CBLB | LCK | GATA1 | ATRIP | NEIL2 | TDP1 | TELO2 |
| ASXL1 | CUL3 | HDAC2 | NKX2-1 | RPTOR | CTR9 | POLQ | IKBKE | CCDC6 | LEF1 | HIST1H3B | FAAP100 | NEIL3 | TDP2 | TIMELESS |
| ATM | CYSLTR2 | HEY1 | NOTCH1 | RUNX1 | CXCR4 | POT1 | MEF2B | CCNB1IP1 | LIFR | KDM5C | FAAP24 | NHEJ1 | TOP3A | TMEM189 |
| ATR | DDR2 | HGF | NOTCH2 | SDC4 | CYLD | PRDM9 | NFKBIA | CD79A | MAPK1 | MAP3K1 | FAAP20 | NUDT1 | TOP3B | WDR48 |
| AURKA | DICER1 | HOOK3 | NPM1 | SDHC | DDB2 | PRF1 | PIK3CD | CD79B | MED12 | KMT2C | MPLKIP | NABP2 | TOPBP1 | GFI1 |
| AXL | DNMT3A | HRAS | NRAS | SERPINB3 | DIS3L2 | PRKAR1A | SRC | CDH11 | NAB2 | NCOR1 | CCNH | OGG1 | TP53BP1 | CYP17A1 |
| B2M | DPYD | IDH1 | NRG1 | SETD2 | DKC1 | PRSS1 | BTG1 | CHD4 | NCOR2 | PHF6 | CDK7 | PARP1 | TREX1 | ELF3 |
| BAP1 | EGFR | IDH2 | NTRK1 | SF3B1 | DOCK8 | PTPN11 | DIS3 | CLIP1 | NDRG1 | PPP2R1A | CETN2 | PARP2 | TREX2 | SGK1 |
| BAZ1A | EPHA2 | IGF1R | NTRK2 | SH2B3 | DROSHA | PTPN13 | EED | CLTCL1 | NONO | PRDM1 | CHAF1A | PARP3 | UBE2A | GSTT1 |
| BCL2 | EPHA3 | IGF2 | NTRK3 | SLC29A1 | ELANE | RAD51B | GNA13 | CNBP | PAX3 | SOCS1 | CLK2 | PCNA | UBE2B | AEN |
| BCOR | ERBB2 | IL7R | PAK1 | SMAD4 | EPCAM | RAD51D | NT5C2 | CNOT3 | PAX7 | SOX9 | DCLRE1A | PNKP | UBE2N | CCNO |
| BLM | ERBB3 | INPP4B | PALB2 | SMARCA1 | ERCC3 | RECQL | PPP2R2A | CREB3L1 | PAX8 | TRAF7 | DCLRE1B | POLB | UBE2T | CENPX |
| BMP5 | ERBB4 | ITGAV | PAX5 | SMARCA4 | ERCC5 | RECQL4 | NSD2 | CREB3L2 | PER1 | IKZF1 | DCLRE1C | POLI | UBE2V2 | CUL4A |
| BRAF | ERCC1 | JAK1 | PBRM1 | SMARCB1 | ETV6 | RFWD3 | EPHA7 | CREBBP | PICALM | MYCL | DDB1 | POLK | UNG | CUL5 |
| BRCA1 | ERCC2 | JAK2 | PDCD1LG2 | SMO | EXT1 | RHBDF2 | GLI1 | CRTC1 | PIM1 | NCOA3 | DMC1 | POLL | USP1 | DNTT |
| BRCA2 | ERCC4 | JAK3 | PDGFB | SRGAP3 | EXT2 | SBDS | MYB | CTCF | POU2AF1 | CDK2 | DUT | POLM | XAB2 | ELOA |
| BRIP1 | ERCC6 | JUN | PDGFRA | SRSF2 | FAH | SDHA | NRG3 | CUX1 | POU5F1 | LATS1 | EME1 | POLN | XRCC1 | HUS1B |
| BTK | EREG | KCNJ5 | PDGFRB | STAG2 | FANCD2 | SDHAF2 | NUP93 | DAXX | PPP6C | LATS2 | EME2 | PRKDC | XRCC3 | PER2 |
| CARS | ESR1 | KDR | PDPK1 | STK11 | FANCE | SDHB | PTK2 | DDIT3 | PRDM16 | YAP1 | ENDOV | PRPF19 | XRCC4 | PER3 |
| CBL | EWSR1 | KIT | PIK3CA | SUZ12 | FANCF | SDHD | RXRA | DDX10 | PREX2 | TEAD2 | ERCC8 | RAD1 | XRCC5 | MSH5 |
| CCND1 | EZH2 | KMT2A | PIK3CB | SYK | FANCI | SERPINA1 | SMARCA2 | DDX3X | PRKACA | MGA | EXO1 | RAD18 | XRCC6 | PARP4 |
| CCND2 | FAM135B | KMT2D | PIK3R1 | TBX3 | FANCL | SETBP1 | TYK2 | DDX5 | PTPRT | HES1 | FAN1 | RAD23A | ABRAXAS1 | POLE3 |
| CCND3 | FAM47C | KRAS | PIK3R2 | TCF3 | FANCM | SH2D1A | ZNF750 | DDX6 | QKI | KDM5A | FANCB | RAD23B | FRK | PPP4R2 |
| CCNE1 | FANCA | LASP1 | PLCG2 | TERT | FAS | SHOC2 | ABI1 | DNM2 | RAD21 | SPEN | GEN1 | RAD52 | BIRC5 | SLX1A |
| CD274 | FANCC | LMNA | PML | TET2 | FEN1 | SLC25A13 | ACKR3 | EBF1 | RANBP2 | THBS2 | GTF2H1 | RAD54B | EMSY | RAD54L2 |
| CDH1 | FANCG | LRP1B | PMS2 | TMEM127 | GALNT12 | SLX4 | ACSL3 | EIF3E | RAP1GDS1 | CUL1 | GTF2H3 | RAD54L | CRKL | RFC5 |
| CDH10 | FAT1 | MAP2K1 | POLD1 | TMPRSS2 | GATA2 | SOS1 | ACVR1 | EIF4A2 | RBM10 | HDAC1 | GTF2H4 | RAD9A | EPHB1 | HMGA2 |
| CDK12 | FBXW7 | MAP2K2 | POLE | TOP2A | GBA | SPOP | AFF4 | ELF4 | RHOA | MLST8 | GTF2H5 | RBBP8 | GLI3 | TSPAN31 |
| CDK4 | FES | MAP2K4 | POLG | TP53 | GJB2 | SPRTN | AMER1 | ELK4 | RHOH | PIK3R3 | H2AFX | RDM1 | IRS2 | MYOD1 |
| CDK6 | FGF19 | MCL1 | PPARG | TPMT | GPC3 | SRY | ARID2 | ELL | RNF213 | RHEB | HELQ | RECQL5 | RUNX1T1 | CHD1 |
| CDKN1A | FGF3 | MDM2 | PPM1D | TSC1 | GREM1 | STAT3 | ATP1A1 | EP300 | SFPQ | RPS6KB1 | HFM1 | REV1 | SLIT2 | ZBTB16 |
| CDKN1B | FGF4 | MDM4 | PRCC | TSC2 | HFE | SUFU | ATP2B3 | EPAS1 | SLC34A2 | GRB2 | HLTF | REV3L | SOX2 | PCDH9 |
| CDKN2A | FGFR1 | MECOM | PRKCH | U2AF1 | HMBS | TGFBR1 | ATRX | EPS15 | SLC45A3 | RIT1 | HMGB1 | RIF1 | SPTA1 | PLXNA1 |
| CDKN2B | FGFR2 | MET | PSIP1 | UGT1A1 | HNF1A | TGFBR2 | AXIN1 | ERC1 | SMAD2 | RASA1 | HUS1 | RMI1 | ZNF217 |  |
| CDKN2C | FGFR3 | MITF | PTCH1 | USP6 | ITK | TP63 | BCL10 | ETNK1 | SMAD3 | ERRFI1 | UVSSA | RMI2 | ZNF703 |  |

**Supplementary Materials and Methods**

**Clinical specimens**

The Formalin-Fixed Paraffin-Embedded (FFPE) tissues samples and blood samples from 5260 CRC patients who have underwent next-generation sequencing (NGS) in a laboratory accredited by the College of American Pathologists (CAP) and Clinical Laboratory Improvement Amendment (CLIA) (3D Medicines Inc., Shanghai, China) from June 2015 to October 2020 were analyzed. Formalin-fixed paraffin-embedded (FFPE) tissue sections were evaluated for tumor cell content using hematoxylin and eosin (H&E) staining. Only samples with a tumor content of ≥20% were eligible for subsequent analyses. The written informed consent was obtained from all included patients. Gene sequencing methods were performed as previously described, including tissue processing and genomic DNA extraction, library preparation and targeted capture (for whole exome sequencing (WES)), library preparation and targeted capture (for tissue-based targeted panel sequencing), DNA sequencing, data processing, and variant calling (for tissue-based testing), and MSI, TMB evaluation [5-8]..

**Tumor immune microenvironment (TIME) by multiplex immunofluorescence (mIF)**Multiplex immunofluorescence staining was conducted using the PANO 7-plex IHC kit (Panovue). Primary antibodies targeting CD8 (clone C8/144B), CD56 (clone 123C3), HLA-DR (clone EPR3692), CD68 (clone BP6036) and PanCK (Cocktail) were sequentially applied to FFPE tissue slides, followed by incubation with horseradish peroxidase-conjugated secondary antibody and tyramide signal amplification. The slides were heat-treated in a microwave after each round of tyramide signal amplification. Cell nuclei acids were stained with 4′-6′-diamidino-2-phenylindole (DAPI, SIGMA-ALDRICH) once all immune cells had been labelled. Multiplex stained slides were scanned using a Mantra workstation configured to capture fluorescent spectra at 20 nm wavelength intervals from 420 nm to 720 nm with a fixed exposure time and an absolute magnification of ×200 and ×100. All scans for each slide were then superimposed to obtain a single image. Besides, PD-L1 expression by immunohistochemistry (IHC) 22C3 antibody.

Images of unstained and monoplex stained lides were used to subtract the spectrum of each fluorophore and tissue autofluorescence respectively. They were also used to create a spectral library required for multispectral unmixing using the inForm Image Analysis Software (PerkinElmer). Slide images were reconstructed without autofluorescence using this spectral library. The quantity of CD8^+^ T cells, macrophages and natural killer cells were expressed as the number of stained cells per square millimeter and as the percentage of positively stained cells in all nucleated cells.

**Statistical analysis**

Data were analyzed using the GraphPad Prism software (version 7.01). Data were presented as the mean ± standard error of the mean (SEM). Differences between two groups were analyzed using the student unpaired t test or an unpaired t test with Welch’s correction. analysis of variance was used to investigate more than two groups.
